# Supplementary material for: The Identification of a Yield-Related Gene Controlling Multiple Traits Using GWAS in Sorghum (Sorghum bicolor L.)
Source: Plants (Basel). 2023 Apr 4;12(7):1557. doi: 10.3390/plants12071557 (PMC10097259; doi:10.3390/plants12071557)
Supplement: Supplementary file 1 [file plants-12-01557-s001.zip › Supplementary Data.pdf]

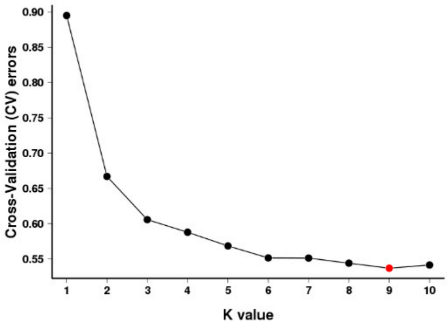

Figure S1. The CV error computed by Admixture.

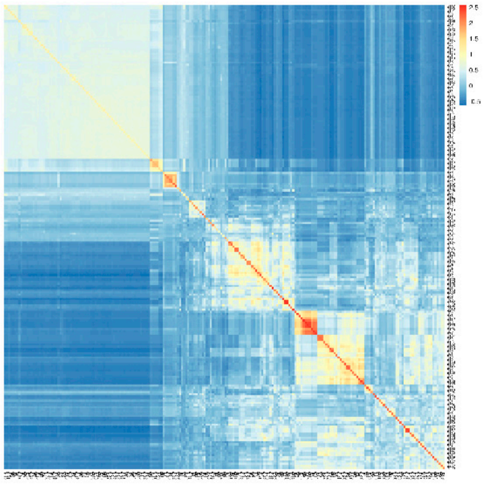

Figure S2. The kinship of 214 landraces based on TASSEL5 analysis.

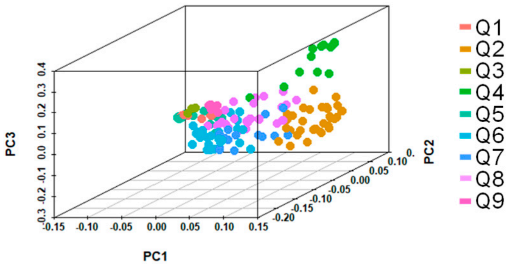

Figure S3. Principal component analysis of sorghum association panel used based on the 2,811,016 SNPs. Different color of each landrace meant different subpopulation based on Admixture.

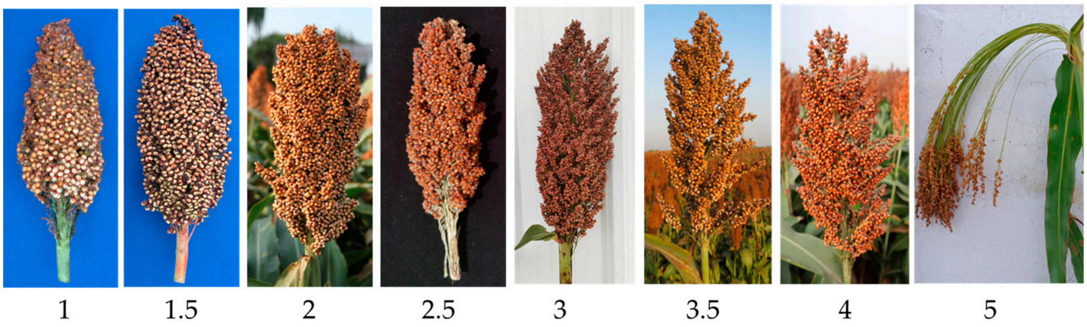

Figure S4. The spike type in the study. 1: Compact; tightly packed branches with a very hard feel in the hand, stinging sensation of hands. 1.5: Compact, tightly packed branches, Not a strong feeling of stinging hand. 2: Semi- compact; the branches are not tightly packed. 2.5: Semi- compact; the branches are not tightly packed. No hard feeling in hand. 3: Semi- loose; branch not dense, side view can transmit light. 3.5: Semi- loose; branch not dense, shorter， partly dispersed. 4: Drooping; branch not dense, completely dispersed 5: Broom shaped; branch side bend.

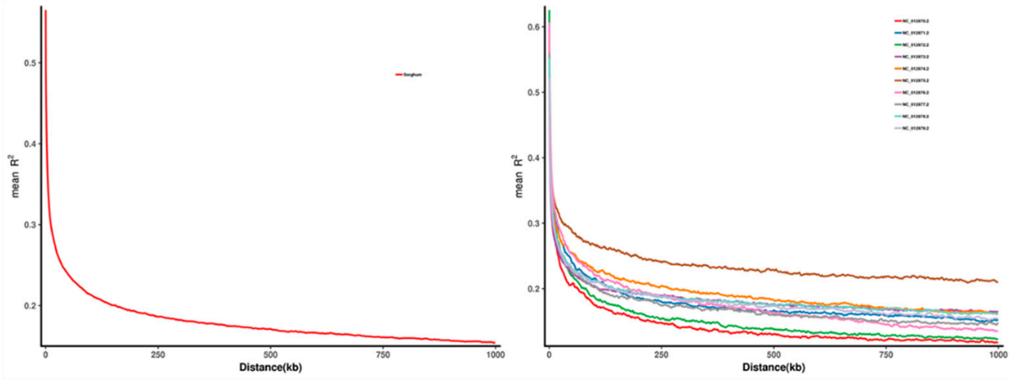

Figure S5. The LD decay of the population (left) and each chromosome (right) based on R^2.

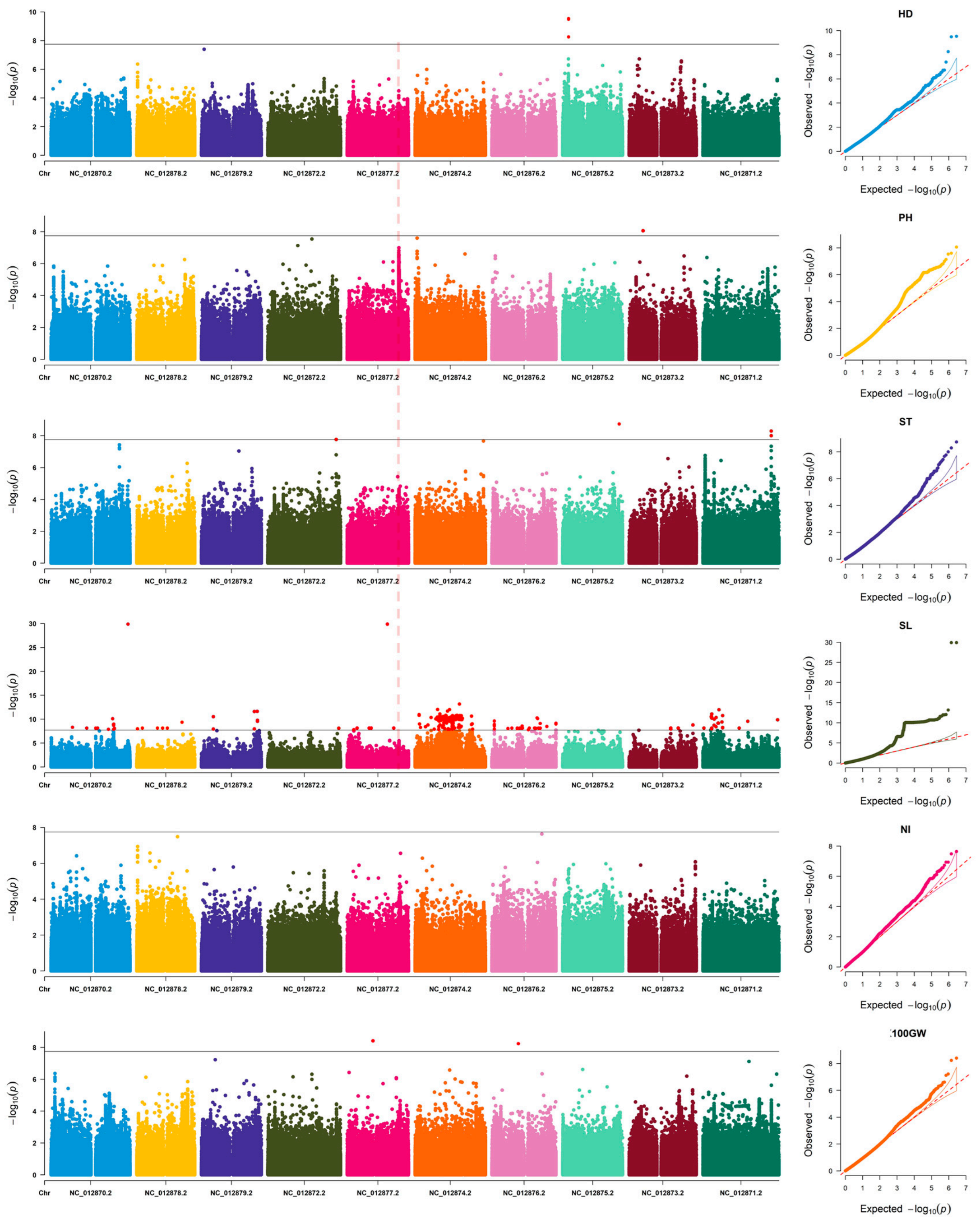

Figure S6. The Manhattan and Q-Q plot of six traits including heading date (HD), plant height (PL), spike type (ST), spike length (SL), number of internodes (NI), and 100-grain weight (100GW) based on the Fast-lmm algorism. The light red dash line indicated the QTL which was identified in four traits.

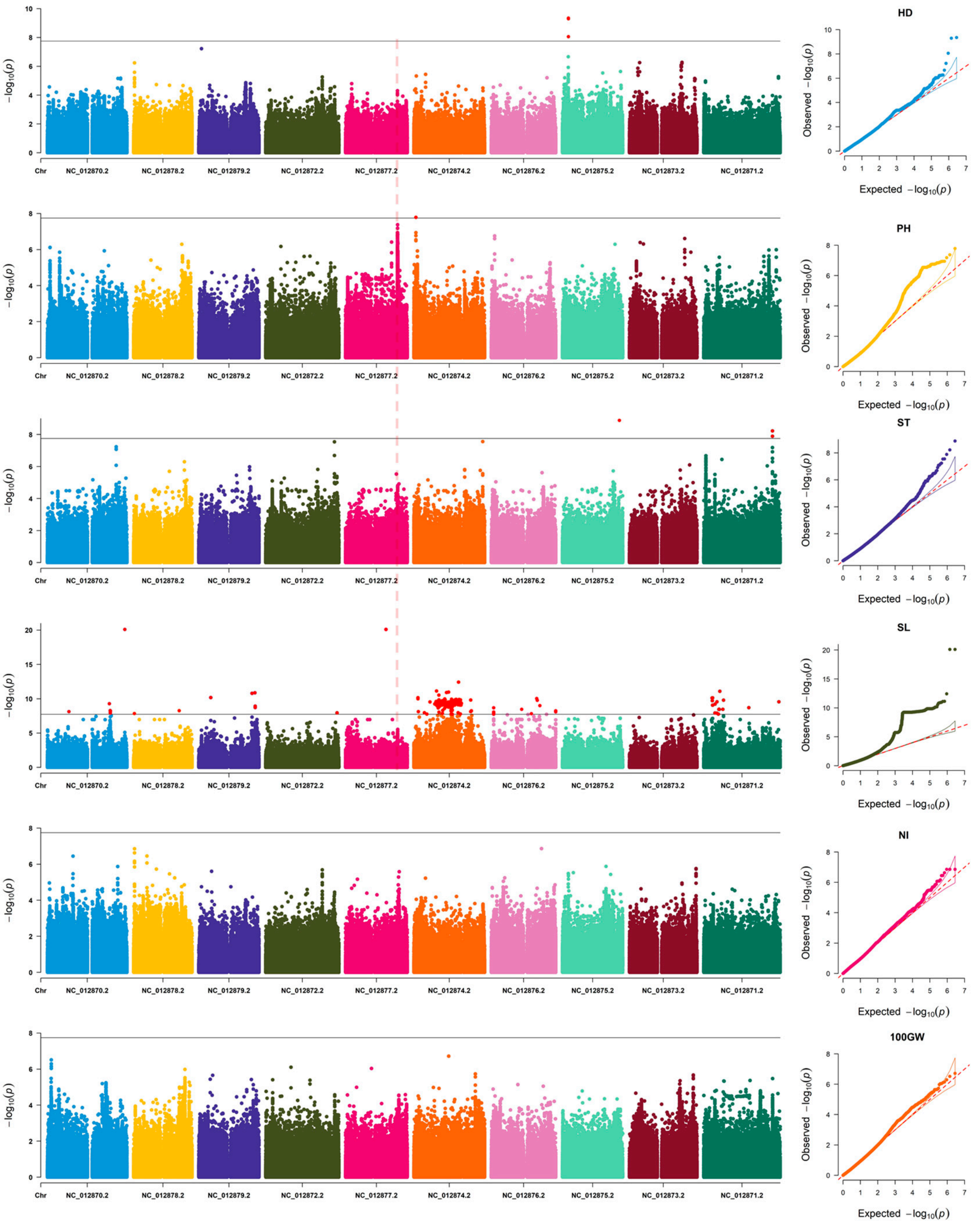

Figure S7. The Manhattan and Q-Q plot of six traits including heading date (HD), plant height (PL), spike type (ST), spike length (SL), number of internodes (NI), and 100-grain weight (100GW) based on the Lmm algorism. The light red dash line indicated indicates the QTL which was identified in four traits.
